# The relationship between the high-risk disordered eating and social network navigation among Saudi college females during the COVID pandemic

**DOI:** 10.3389/fpubh.2022.949051

**Published:** 2022-09-06

**Authors:** Alotaibi Abdulaziz Raja N, Nermin A. Osman, Abdullah Muidh Alqethami, Nesrin Kamal Abd El-Fatah

**Affiliations:** ^1^Department of Public Health, Directorate of Health Affairs in Taif, Ministry of Health, Riyadh, Saudi Arabia; ^2^Department of Biomedical Informatics and Medical Statistics, Medical Research Institute, Alexandria University, Alexandria, Egypt; ^3^Department of Nutrition, High Institute of Public Health, Alexandria University, Alexandria, Egypt

**Keywords:** Saudi Arabia, college, social network navigation, high-risk disordered eating, female, social media, COVID pandemic

## Abstract

**Background:**

Disordered eating behaviors (DEBs) are complex health issues that may lead to negative physical and mental health outcomes among college students. More studies should be directed toward the screening of DEBs. This study aimed to determine the prevalence of DEBs among Saudi female university students and their association with social networking site (SNSs) usage and composite lifestyle behaviors during the unprecedented period of COVID-19.

**Methods:**

This cross-sectional study included 445 females recruited using stratified random sampling. The participants self-reported demographic, social, medical, and lifestyle data and completed the validated Arabic version of the Eating Attitudes Test-26, Social Networking Sites (SNSs) Usage Questionnaire, Bergen Social Media Addiction Scale, and Body Shape Questionnaire.

**Results:**

The prevalence of DEBs was 27.2% among the female students at Taif University. From the pre-pandemic period until the current time, the DEBs-risk group had a significantly higher SNS navigation rate (36.4%) than the normal group (20.4%) (*X*^2^ = 30.015, *p* = 0.001). The regression analysis revealed that females with a significant body image concern, higher number of SNSs friends, and frequent visits to SNSs, and those seeking social-dependent information in relation to weight loss/dieting were more likely to develop DEBs (Overall Model: Chi-Square *X*^2^ = 158.071, *p* < 0.000^**^).

**Conclusions:**

SNSs usage and DEBs were associated during the COVID-pandemic. However, the composite lifestyle score did not demonstrate a significant association with DEBs among the female students at Taif University. Investigating the magnitude of DEBs and understanding the role of SNS are essential for preventing disordered eating among young females.

## Introduction

Disordered eating behaviors (DEBs) or high-risk disordered eating refers to problematic eating habits that are less severe in their behavioral manifestations than those required to meet the full criteria for the diagnosis of an eating disorder (ED) on the basis of the Diagnostic and Statistical Manual of Mental Disorders, Fifth Edition (DSM-5). It includes unhealthy eating habits such as fasting, restrictive dieting, skipping meals, compulsive overeating, unbalanced eating, vomiting, misuse of laxatives, diuretics, enemas, and the use of diet pills to lose weight (1).

DEBs are proven risk factors for EDs. Dieting and other obsessive weight control practices, fears of fatness, negative body image, and intensive food and weight preoccupation are types of eating impairments associated with an increased risk of developing anorexia and bulimia nervosa (2). In 2019, a systematic review of population-based studies revealed that EDs increases the vulnerability to psychiatric illnesses, diabetes, weight fluctuations, poor nutritional intake and quality, obesity, suicide, and other forms of premature mortality (3).

The prevalence of EDs ranged from 4.6% in the U.S. to 3.5% in Asia and 2.2% in Europe (4, 5). The prevalence of clinically diagnosed EDs is low; however, DEBs are prevalent among college-aged adolescents and young females (6). In a recent systematic review and meta-analysis conducted in the Middle East (16 countries including Saudi Arabia), the overall prevalence rate of DEBs was 22.07%, which was relatively higher than the global prevalence rate (7). Previous studies on female Saudi university students using the EAT-26 tool revealed that 35.4, 29.4, and 25.4% of them, demonstrated DEBs (8–10).

In a recent study conducted during the COVID-19 pandemic (2022), out of 1004 Saudi females, approximately 31.5% were at risk of developing EDs (11). Studies on DEBs-associated factors have focused mainly on socio-demographic factors (e.g., college-aged females), sociocultural factors (e.g., perceived pressure from family and peers), lifestyle factors, social media, concerns about body image, society's thin ideal, weight status, personality traits, as well as psychological, genetics, and biological factors (12–14). In addition, female university students who have DEBs are reported to have been under family pressure to lose weight, overweight or obese, married, physically active, studying in health science colleges, and with poor eating habits (8–11). In Saudi Arabia, youth aged 15–34 account for approximately one-third of the population, and approximately 50% are females (15). The prevalence of risky lifestyle behaviors is very high among young females. According to the National Saudi Health Information Survey including 2,382 youths, the prevalence of overweight or obesity, physical inactivity, and daily consumption of fewer than five servings of fruits and vegetables among Saudi young females was 43.9, 75.6, and 92%, respectively (16). In Taif university, 55% female students were overweight and obese, 9 % smoked cigarettes, 35% consumed vegetables and fruits lower than the recommended amounts, and 38 % used social media for 3 h or more per day (17).

Social networking sites (SNSs) are web-based services that enable users to create public or semi-public profiles and share connections with a detailed list of other users. SNSs have attracted the interest of many adolescents and young adults and have become a novel area of research. Females use SNSs more than men for various social purposes (18, 19), and females tend to be more likely to use SNSs to compare themselves with others and search for information (18). Recently, Aldakhil et al. (14) studied 763 university students in Jeddah, Saudi Arabia, and found that social media significantly affected females at risk of EDs more than men. As a response to current Western sociocultural influences, the increasing use of SNSs such as Facebook, Twitter, and Instagram offers numerous opportunities to promote beauty ideals, social comparisons, and the drive for thinness among female college students (13, 20). Consequently, it is negatively related to body satisfaction, leading to DEBs (21). A recently published meta-analysis involving 13,301 respondents revealed a positive correlation between the use of SNSs and DEBs (22). Nonetheless, a few studies reported no correlation between them (23, 24).

During the critical period of the COVID-19 pandemic and the resulting mandated social isolation measures, several studies revealed a significant impact of increased SNSs utilization for socialization and keeping up with local and global events on population mental health and lifestyle behaviors (25–27). According to the Internet world statistics released in 2021, 90.1% of the Saudi Arabian population uses the Internet. Research from the Global Web Index indicates that the amount of time Saudi Arabians spent on social media has increased by 25% from 2017 to 2021. In 2021, Saudis spent 196 min per day on social media on an average, 10 min more than that in 2019 (28).

Given that DEBs may develop into EDs with severe consequences, it is crucial to properly define DEBs, their risk factors, and their impact on general health so that preventive measures can be devised. Previous studies have found some discrepancies in the reported relationships between the duration spent on social media and DEBs (22, 29, 30), but they rarely considered the potential effects of SNS-affective experiences that may be reflected on the psychological well-being (31). Disordered eating may be associated with lifestyle factors such as physical activity, poor eating habits, smoking, sleep disturbance, and obesity (8–11). In addition, the clustering of unhealthy lifestyle behaviors has synergistic and more detrimental effects on health than the individual effects of health behaviors (32). Accordingly, we hypothesized that high-risk DEBs among Saudi female university students would be associated with SNSs usage, composite lifestyle behaviors, and self-perception of body image during the unprecedented COVID-19 pandemic period.

## Subjects and methods

### Design and study population

A cross-sectional study was carried out in female students at the University of Taif, Saudi Arabia, between January 17 and 30 during the academic year 2021–2022. Using the EPI-INFO 2002 software, the minimum sample requirement was determined to be 351, based on the prevalence rate of DEBs among female students (35.4%) determined using the EAT-26 tool that had a precision of 3% and a confidence level of 95%. Our total sample size was 445; we increased the sample size to more than 20% to minimize the sampling error. A multistage stratified random sampling technique was used to obtain a sample that best represents the entire population, ensuring that each subgroup of interest was represented (33). Stratification was based on the type of faculty (practical vs. theoretical) and grade level. Four out of 13 faculties of Taif University (nearly one-third of the 13 faculty members) were selected randomly through the lottery method (two practical and two theoretical faculties). The predetermined sample was proportionally allocated to the selected faculties: Faculty of Medicine (45/666 students), Faculty of Engineering (24/353 students), Faculty of Literature (152/2,264 students), and Faculty of Sharia and Regulation (224/3,353 students). In the subsequent stages, samples from each faculty were assigned equally to all the grades. In the third stage, a predetermined sample from each grade was collected from a randomly selected class. Females with a history of diagnosis or treatment of EDs and those affiliated with university branches outside Taif city were excluded. The self-administered paper-based questionnaires were delivered to the participants during the class activities to share in the study under the supervision of the main author for clarification and to ensure that there was no side talk and to avoid socially expected answers. Therefore, it is noteworthy to mention that the response rate was 96%; 10 questionnaires were excluded because of incompleteness. This study was conducted according to the guidelines laid down in the Declaration of Helsinki and all the procedures involving the research study participants were approved by the Research Ethics Committee of Taif Health Affairs, Ministry of Health, Saudi Arabia approved this study (IRB. HAP-02-T-067, 165). Written consent was obtained from all the participants before answering the questions, and confidentiality was assured.

### Measures

Female students self-reported their demographic, medical, lifestyle, weight, and height information and completed validated questionnaires based on DEBs [Eating Attitudes Test-26 [EAT-26] (34), SNSs Usage (35), Bergen Social Media Addiction Scale (BSMAS) (36), and Body Shape Questionnaire (37)].

#### Eating attitudes test-26: EAT-26

The EAT-26 is a screening tool used worldwide to identify individuals who present attitudes associated with abnormal eating behaviors or those at risk of developing EDs (5, 38–40). It has been established as a reliable and valid instrument in Arabic (Cronbach's α = 0·89) (41). Additionally, the Arabic version of the EAT-26 has been validated among female students in Saudi Arabia, and the overall reliability of the Arabic version of the EAT-26 was assessed with Cronbach's α = 0.83. The EAT-26 comprises 26 questions, and each one has six possible answers ranging from “infrequently/almost never/never” [0/1/2] to “always” [3] (42). This scale includes three subscales: dieting, bulimia/food preoccupation, and oral control. It also includes four additional behavioral questions that assess self-reported binge eating, self-induced vomiting, use of laxatives or diuretics, and treatment of EDs. Females who scored ≥20 or answered affirmatively to any of the behavioral questions were classified as being at risk of EDs, and higher scores indicated a greater risk of EDs (34).

#### Social networking sites usage questionnaire

The questionnaire included questions on featured and affective SNSs usage. The featured subscale included 13 items assessing basic (questions on the frequency of use, the average extent of time of use, and the number of friends), interactive (frequency of sending messages, updating status, sharing or resending profiles, visiting a friend's homepage, and commenting on others' photos and comments), and self-display usage (writing notes/blogs, updating profile images, and posting photos) on a 7-point scale (1 = never, 7 = multiple times a day). For the affective SNSs usage items, participants rated the frequencies of experiencing eight negative and positive emotions using a scale ranging from 0 to 7, where higher scores indicated higher usage or frequency of emotions. The measure has been validated in young adults with good internal consistency (α = 0.82). Three questions were added to explore exposure the type and duration of social networks use including No 0) or Yes 1) responses to the following question “Please indicate which of the following accounts you have?” (Twitter, Snap Chat, Facebook, YouTube, WhatsApp, and Instagram); in the context of the most used SNSs in Saudi Arabia, “Which social networking site do you use the most?” and “When did you create your first account on social networking sites?” (35). We asked about the change in SNSs navigation rate from pre-pandemic stage to current time by a three-scale question Yes, I navigate it less than before 1), Yes, I navigate it more than before 2), and No change 0).

Some questions related to SNSs usage to fulfill specific gratifications related to weight loss/dieting, fitness/exercise, cooking, fashion, and bariatric surgery were added. These values were adapted from Park et al. (43) and Lee et al. (21). They included three types of SNSs use: information-seeking (e.g., “I visit SNSs to gather information about weight loss/dieting, cooking, fashion, bariatric surgery, and fitness/exercise,” with a dichotomous (i.e., yes or no) response for each; self-status seeking (e.g., “In the past 3 months, I posted messages on my own SNS with the intent to express my ideas and opinions about weight loss/dieting, fitness/exercise, cooking, fashion, bariatric surgeries,” with a dichotomous (i.e., yes or no) response for each); and socializing use (for example, “On an average, how many messages or comments do you post on others' posts with a desire to interact with another individual about weight loss/dieting, fitness/exercise, cooking, fashion, and bariatric surgeries?,” with six responses varying from never (0) to many times per day (7) (21). The frequencies for each type of SNSs use were summed to determine each participant's general tendency to use SNSs for information seeking, self-status seeking, and socializing use. In addition, the participants answered a question related to why they used SNSs in relation to weight loss/dieting, fitness/exercise, cooking, fashion, and bariatric surgery for checking the appropriate response. Responses ranged from very rarely (1) to very often (5), and the scores ranged from 11 to 55. Higher scores indicated higher social motives to use social media for weight loss/dieting, fitness/exercise, and body appearance (21, 43). The Networking SNSs Usage Questionnaire demonstrated good reliability with Cronbach's α = 0.813 for featured use, 0.76 for affective use, and 0.82 for the dependence scale.

#### Dependence on social networking sites

SNSs dependence was assessed using the Bergen Social Media Addiction Scale (BSMAS). It is a valid and reliable questionnaire which determines the use of social media activities generally rather than to one specific platform (36). It consists of six items that indicate addiction criteria such as withdrawal, salience, mood modification, conflict, tolerance, and relapse. It is rated on a five-point scale ranging from strongly disagree (1) to strongly agree (5), with scores ranging from 11 to 55. A score of 24 is set as the clinical cut-off point based on the gold standard for clinical diagnosis (44). The validated Arabic questionnaire demonstrated good reliability, with a Cronbach's α of 0.754.

#### Body shape questionnaire (BSQ-8)

The BSQ-8 is an 8-item scale with six response options on a Likert scale ranging from never = 1 to always = 6 (37). It showed adequate reliability among females, as evaluated by Cronbach's alpha (α = 0.91), to evaluate body image problems such as fears of weight gain, desires for weight loss, body shape concern, and low self-esteem due to one's physical appearance was used to assess body shape concerns. Item responses were summed up. A score of less than 19 indicates no concern with shape; a score of 19–25 indicates moderate concern with shape; and a score of more than 33 indicates severe concern with shape (45). The validated Arabic questionnaire demonstrated good reliability, with a Cronbach's α of 0.78.

### Adoption and validation procedures

These procedures were conducted on 50 students for all questionnaires except the EAT-26. According to the Guidelines of Beaton et al. (46), forward translation was initially performed by two native Arab bilingual translators who were fluent in English. A backward translation was then performed by two native English-speaking translators who were fluent in Arabic and unfamiliar with the concepts of the scales. The back-translated English questionnaire was subsequently compared with the original English questionnaire, and inconsistencies between the two versions were resolved to ensure that the translation did not affect the content validity of the questionnaire.

Content validity was assessed to ensure the necessity of each item in the collected pilot using qualitative and quantitative methods by a five-expert panel consisting of a psychologist, two statisticians, a nutritionist, and a public health specialist. For the qualitative evaluation, we submitted only the Arabic translation without substitution. Each item was double-checked by two volunteer translators for the correct wording to ensure that the items were free of any ambiguous wording that could confuse the students. For the quantitative measurement of content validity, content validity index (CVI) and content validity ratio (CVR) were calculated holistically. CVI (0.90; range [0.86–1]) and CVR [0.85; range (0.80–1)] were both satisfactory.

#### Composite lifestyle index

Five lifestyle behaviors (physical activity, sleep, sitting, smoking, Body Mass Index (BMI) in kg/m^2^, and dietary habits) were assessed based on reported risk category calculation protocols (47). Dietary habits were measured based on index values reported by the Saudi Food-based Dietary Guidelines for 14 food items (48). The participants reported the number of fruit and vegetable servings per day; three is the optimal number of servings. The weekly frequencies of the remaining 12 food items were assessed, which included full-fat dairy products; non-refined cereals and bread; legumes and nuts; fish and seafood; red meat and other meat products; poultry; butter or margarine; fast foods; sweets; potato chips or French fries; sugar-sweetened drinks/soft drinks; and, energy drinks. **Five** options for eating frequency were established: “daily;” “5–6 times a week;” “3–4 times a week;” “1–2 times a week,” and “never or rarely.” The responses ranged from 0–4 (for food items recommended in the Saudi dietary guidelines) or the reverse (for food items that should be limited in the Saudi dietary guidelines). The total scores ranged from 0 to 56. The total score was subsequently classified into three tertiles using the following equation (first tertile = lower limit (11) + 0.33 × 32= 21.56, second tertile= lower limit (11) + 0.66 × 32 = 32.12). A score of one was generated for those below 21.56 (first level tertile or poor diet) and from 21.56 to less than 32.12 (second level tertile or average diet); a score of zero was generated for those above 32.12 (third level tertile or better diet). These tertiles were subsequently classified into low-risk (0 = third tertile) and high-risk (1 = first and second tertiles), based on a previous study (49). Sleeping, sitting, and smoking behaviors were dichotomized into healthy (low-risk) and unhealthy (high-risk) categories and scored as 1 and 0, respectively, whereas physical activity (PA) was scored as 0 (high PA), 1 (moderate PA), and 2 (low PA). Finally, BMI was calculated and categorized: 1 (underweight/overweight/obese) and zero (normal BMI). The scores for the five behaviors were added.

### Statistical analysis

Statistical analyses were performed using IBM (SPSS) Statistics version 24.0^*^ software. Descriptive statistics, including frequencies and percentages, were used for categorical variables, and median and range were used for continuous variables after determining normality using the Shapiro test. The rates of healthy and unhealthy dichotomies were calculated for each lifestyle behavior, and the rates of the participants engaging in one to seven unhealthy lifestyle behaviors were calculated. BMI (kg/m^2^) was computed based on the given weight and height and classified according to the World Health Organization guidelines.

Chi-squared test was used to compare the DEBs categories, and Monte Carlo exact test was used in case of violation of the chi-square assumption. Spearman's rho correlation coefficients were determined to test the association between the continuous variables, and Mann–Whitney *U*-test was used to test the difference in the motivation score between normal group and those at risk. A logistic regression model for the DEBs variable (dummy variable) was used to determine significant contributors. For all the statistical tests, the significance level was determined to be below 5% and quoted as a two-tailed hypothesis test.

## Results

Totally, 445 university female students were included in the study and classified according to the EAT-26 cutoff values into the normal group (*n* = 324, 72.8%) and DEBs-risk group (*n* = 121, 27.2%). Approximately half of them were affiliated with the College of Sharia and Regulations (50.3%), and the rest were affiliated with Faculty of Literature (34.2%), Faculty of Medicine (10.1%), and Faculty of Engineering (5.4%). The majority were single, living with their families, and not working outside the study period; the median age was 21 years. The socioeconomic status did not differ significantly between the normal and DEBs-risk groups. Regarding the medical status, the DEBs-risk group showed a significantly higher rate of psychological illnesses, regular medication intake, and familial history of obesity than the normal group (proportional differences = 8.1, 7.2, and 14.5%, respectively, *p* < 0.05, Table 1). A simple frequency table of the items of the EAT-26 is summarized in Supplementary Table S1.

**Table 1:** Sociodemographic characteristics among female university students, classified according to EAT-26.

	**Total**	**EAT**−**26**		**Statistical test**	***p***
	***N* = 445**	**Normal**	**At Risk**		
		**(*n* = 324 ,72.8%)**	**(*n* = 121, 27.2%)**		
Faculty:					
Faculty of Medicine	45 (10.1%)	36 (11.1%)	9 (7.4%)		
Faculty of literature	152 (34.2%)	111 (34.3%)	41 (33.9%)	2.254	0.521^a^
Faculty of Engineering	24 (5.4%)	19 (5.9%)	5 (4.1%)		
College of Sharia and Regulations	224 (50.3%)	158 (48.7%)	66 (54.6%)		
Age (years)				1.928	0.381^a^
<19 years	111 (24.9%)	84 (25.9%)	27 (22.3%)		
19–21	140 (31.5%)	96 (29.6%)	44 (36.4%)		
22 and more	194 (43.6%)	144 (44.5%)	50 (41.3%)		
Age Median (Range)	21 (17–30)	21 (17–30)	21 (17–27)	−0.318	0.751^c^
Marital Status:					
Single	396 (89.0%)	286 (88.3%)	110 (90.9%)	1.836	0.593 ^b^
Married (Non-Pregnant)	34 (7.6%)	27 (8.4%)	7 (5.8%)		
Married (Pregnant)	8 (1.8%)	5 (1.6%)	3 (2.5%)		
Divorced	7 (1.6%)	6 (1.7%)	1 (0.8%)		
Residence				1.681	0.62 ^b^
Living with my family	424 (95.3%)	309 (95.4%)	115 (95.0%)		
Living with foreign	7 (1.6%)	6 (1.8%)	1 (0.8%)		
Living alone in a private home	10 (2.2%)	7 (2.2%)	3 (2.5%)		
Living with relatives or friends	4 (0.9%)	2 (0.6%)	2 (1.7%)		
Working outside the study time?				1.561	0.514 ^b^
No	397 (89.2%)	290 (89.5%)	107 (88.4%)		
Yes, Partial Time	28 (6.3%)	18 (5.6%)	10 (8.3%)		
Yes, Full time	20 (4.5%)	16 (4.9%)	4 (3.3%)		
Father Work				4.589	0.309 ^b^
Not working	24 (5.4%)	20 (6.2%)	4 (3.3%)		
Governmental Sector Employer	159 (35.7%)	109 (33.6%)	50 (41.3%)		
Free Lancer	45 (10.2%)	34 (10.5%)	11 (9.1%)		
Private Sector Employer	21 (4.7%)	17 (5.2%)	4 (3.3%)		
Retired	183 (41.1%)	133 (41.0%)	50 (41.3%)		
Died	13 (2.9%)	11 (3.5%)	2 (1.7%)		
Mother Work				3.747	0.125 ^b^
Housewife	293 (65.8%)	216 (66.7%)	77 (63.6%)		
Governmental Sector Employer	119 (26.7%)	88 (27.2%)	31 (25.6%)		
Student	13 (2.9%)	7 (2.2%)	6 (5.0%)		
Private Sector Employer	16 (3.7%)	11 (3.4%)	5 (4.2%)		
Died	4(0.9%)	2 (0.5%)	2(1.6%)		
Father Education				4.003	0.678 ^b^
Illiterate	16 (3.6%)	10 (3.1%)	6 (5.0%)		
Primary	50 (11.2%)	36 (11.1%)	14 (11.4%)		
Preparatory	62 (13.9%	50 (15.4%)	12 (10.0%)		
Secondary	123 (27.6%)	91 (28.1%)	32 (26.4%)		
Diploma	19 (4.3%)	12 (3.7%)	7 (5.8%)		
University	138 (31.0%)	98 (30.2%)	40 (33.0%)		
Higher Education	37 (8.4%)	27 (8.4%)	10 (8.4%)		
Mother Education				8.973	0.173 ^b^
Illiterate	64 (14.4%)	46 (14.2%)	18 (14.9%)		
Primary	79 (17.8%)	60 (18.5%)	19 (15.7%)		
Preparatory	37 (8.3%)	31 (9.6%)	6 (5.0%)		
Secondary	88 (19.8%)	60 (18.5%)	28 (23.1%)		
Diplome	16 (3.6%)	14 (4.3%)	2 (1.6%)		
University	136 (30.6%)	99 (30.6%)	37 (30.6%)		
Higher Education	25 (5.5%)	14 (4.3%)	11 (9.1%)		
Family Income				1.735	0.773^a^
5000 or less	116 (26.1%)	89 (27.5%)	27 (22.3%)		
5001–10,000	125 (28.1%)	91 (28.1%)	34 (28.1%)		
10,001–15,000	91 (20.4%)	66 (20.4%)	25 (20.7%)		
15,001–20,000	56 (12.6%)	38 (11.7%)	18 (14.9%)		
20,001 or more	57 (12.8%)	40 (12.3%)	17 (14.0%)		
Social Status for mother and father				6.672	0.074 ^b^
Divorced	28 (6.3%)	20 (6.2%)	8 (6.6%)		
Together	380 (85.4%)	276 (85.2%)	104 (85.9%)		
Both Died	6 (1.3%)	2 (0.6%)	4 (3.3%)		
Father Died	31 (7%)	26 (8.0%)	5 (4.2%)		
Psychological Disease history	51 (11.5%)	30 (9.3%)	21 (17.4%)	5.849	0.016*^a^
Regular medication intake	43 (9.7%)	25 (7.7%)	18 (14.9%)	5.17	0.023*^a^
Family History of Obesity	122 (27.4%)	76 (23.5%)	46 (38.0%)	9.358	0.002*^a^
Family History of psychological disorders	31 (7.0%)	20 (6.2%)	11 (9.1%)	1.157	0.228^a^
Have you ever been infected with COVID (with confirmed Positivity)	154 (34.6%)	120 (37.0%)	34 (28.1%)	3.11	0.07^a^
Family and peer Support concerning Body weight	295 (66.3%)	214 (66.0%)	81 (66.9%)	0.031	0.85^a^

^a^Chi-square test, ^b^Monte-Carlo corrected p-value, ^c^Mann Whitney U-test, ^*^p <0.05.

Table 2 illustrates the lifestyle characteristics and body image concerns of female students. The majority (74.8%) showed a low level of PA, although the DEBs-risk group showed a lower rate of low PA and a higher rate of high PA compared to the normal group (proportional differences = 7.5 and 7.3%, respectively, *p* = 0.018). Half of the students had normal body weights; however, the probability of being overweight and obese was significantly high in the DEBs-risk group (*p* = 0.008). There was a marked increase in the body image-related concern in the DEBs-risk group (37.2%). The normal group had higher proportions of students with no and mild body image concerns and a lower proportion of those with moderate body image concerns compared to the DEBs-risk group (proportional differences = 15.6, 2.9, and 3.2%, respectively, *p* = 0.001). There were no statistically significant differences between the DEBs categories with respect to smoking habits, dietary habits, sleeping habits, and overall composite lifestyle score (*p* > 0.05).

**Table 2:** Lifestyle characteristics and body image concern among female university students, classified according to EAT-26.

	**Total**	**EAT**−**26**		**Statistical test**	***p***
	***N* = 445**	**Normal**	**At Risk**		
		**(*n* = 324 ,72.8%)**	**(*n* = 121, 27.2%)**		
Level of physical activity:					
Low	333 (74.8%)	249 (76.9%)	84(69.4%)	8.010	0.018*
Moderate	84 (18.9%)	61 (18.8%)	23 (19%)		
High	28 (6.3%)	14 (4.3%)	14 (11.6%)		
Sitting time				0.090	0.764
Meeting recommendations	386 (86.7%)	282 (63.4%)	104 (23.4%)		
Sleeping				0.179	0.672
Meeting recommendations	79 (17.8%)	56 (17.3%)	23 (19.0%)		
BMI (kg/m^2^):				11.767	0.008*
Underweight	86 (19.6%)	72 (22.4%)	14(12%)		
Overweight	87 (19.8%)	56 (17.4%)	31 (26.5%)		
Obese	32 (7.3%)	19(5.9%)	13 (11.1%)		
Smoking				1.297	0.255
Non-smoking or Ex-smoker	18 (4.0%)	11 (3.4%)	7 (5.8%)		
Smoker	427 (96.0%)	313 (96.6%)	114(94.2%)		
Optimal fruit intake per day	282 (63.4%)	198 (61.1%)	84 (69.4%)	2.621	0.105
Optimal Vegetables intake /day	418 (93.6%)	303 (93.5%)	115 (95%)	0.358	0.549
Optimal Fast-food intake (Never/rarely)	44 (9.9%)	29 (9%)	15 (12.4%)	1.174	0.279
Sweets (Never/rarely)	22 (4.9%)	16 (4.9%)	6 (5%)	0.001	0.993
Energy drinks	59 (13.3%)	44 (13.6%)	15 (12.4%)	0.107	0.743
French fries	73 (16.4%)	57 (17.6%)	16 (13.2%)	1.227	0.268
Diet risk category					
Lowest tertile (Poorer diet)	78 (17.5%)	62 (19.1%)	16 (13.2%)	2.300	0.317
Middle tertile (Average diet)	321 (72.1%)	228 (70.4%)	93 (76.9%)		
Highest tertile (better diet)	46 (10.4%)	34 (10.5%)	12 (9.9%)		
Composite lifestyle Score:					
1–2 Unhealthy Behavior	20 (4.5%)	12 (3.7%)	8 (6.6%)	2.220	0.333
3–4 Unhealthy Behaviors	256 (57.5%)	191(59.0%)	65 (53.7%)		
5–7 Unhealthy Behaviors	169 (38.0%)	121(37.3%)	48 (39.7%)		
Body image concern					
Mild concern	138 (31.0%)	103 (31.8%)	35 (28.9%)	15.822	0.001*
Moderate Concern	19 (4.3%)	11 (3.4%)	8 (6.6%)		
Marked concern	116 (26.1%)	71 (21.9%)	45 (37.2%)		

^*^p <0.05 was considered significant using chi-square test.

Table 3 illustrates the SNS use among female students. The SNSs accounts of the majority of the students (62%) were created more than 7 years. The Snapchat was used by 82% of the participants, followed by YouTube (70.8%) and TikTok (69.2%). When we asked them to rank how often they used social media, Snapchat came first (27.3%), followed by Tiktok (21.6%), then Instagram (17.8%). There was no association of the DEBs categories with the number of years and the preference for SNSs usage. Moreover, two-thirds (68%) of them visited SNSs (frequency of use is once or more than an hour), and approximately 40% of them spent from 30 min to 3 h in each access, and 30% of the students spent more than 3 h. For featured usage, the DEBs-risk group showed a higher frequency of account checking, duration of use, and number of friends on different SNSs compared to the normal group (*p* < 0.05). For affective usage, unhappiness was significantly linked with risky DEBs (*p* = 0.007). For addictive usage, there was no statistically significant difference between the DEBs categories. However, the median motivation score of using SNSs in relation to weight loss/dieting, fitness/exercise, cooking, fashion, and bariatric surgery was highly significant in the DEBs-risk group (*p* < 0.001). In addition, there was a significant difference in the SNSs usage between the pre-pandemic time and the current time among the DEB-risk group compared to the normal group (proportional differences in the high use, and less use = 16%, 13.1%, respectively, *p* = 0.001). The details of affective, featured use, and addiction of SNSs are displayed in Supplementary Table S2.

**Table 3:** Social network sites use (type, featured usage, affective use, SMD social media disorder and motive of SNSs use) among female university students, classified according to EAT-26.

	**Total**	**EAT**−**26**		**Statistical test**	***p***
	***N* = 445**	**Normal**	**At Risk**		
		**(*n* = 324 , 72.8%)**	**(*n* = 121, 27.2%)**		
Instagram Use	201 (45.2%)	143 (44.1%)	58 (47.9%)	0.379	0.538^a^
Twitter Use	125 (28.1%)	90 (27.8%)	35 (28.9%)	0.012	0.913^a^
WhatsApp Use	40 (9%)	27 (8.3%)	13 (10.7%)	3.535	0.06^a^
Facebook Use	18 (4%)	15 (4.6%)	3 (2.5%)	0.107	0.734^a^
Snapchat Use	367 (82.5%)	273 (84.3%)	94 (77.7%)	2.63	0.105^a^
YouTube Use	315 (70.8%)	222 (68.5%)	93 (76.8%)	2.964	0.085^a^
LinkedIn Use	21 (4.7%)	15 (4.6%)	6 (4.9%)	0.021	0.884^a^
TikTok Use	308 (69.2%)	226 (69.8%)	82 (67.8%)	0.163	0.678^a^
Telegram Use	36 (8.1%)	25 (7.7%)	11 (9.1%)	0.224	0.636^a^
What social networking site do you use the most?				9.934	0.270^b^
Instagram	79 (17.8%)	53 (16.4%)	26 (21.5%)		
WhatsApp	50 (11.3%)	39 (12.1%)	11 (9.1%)		
Facebook	11 (2.5%)	10 (3.1%)	1 (0.8%)		
TikTok	96 (21.6%)	72 (22.2%)	24 (19.8%)		
You tube	42 (9.5%)	30 (9.3%)	12 (10.0%)		
Twitter	43 (9.7%)	30 (9.3%)	13 (10.7%)		
Snapchat	123 (27.3%)	90 (27.3%)	33 (27.3%)		
Telegram	1 (0.3%)	0 (0.0%)	1 (0.8%)		
Holding SNS account (year)				5.303	0.258^a^
Less than 2 years	34 (7.6%)	23 (7.1%)	11 (9.1%)		
More than 10 years	104 (23.4%)	73 (22.5%)	31 (25.6%)		
Featured Usage: 1- Basic SNSs usage factor				
SNS account check (times)				14.942	0.034*^b^
Never	10 (2.2%)	5 (1.5%)	5 (4.1%)		
Extreme use (once or more an hour)	303 (68.1%)	217 (67.0%)	86 (71.0%)		
Duration of using SNSs				17.257	0.008*^b^
15 min or less	39 (8.8%)	31 (9.6%)	8 (6.6%)		
More than 4 h	138 (31.0%)	86 (26.6%)	52 (42.9%)		
Number of friends				19.868	0.003*^b^
1 - <50	285 (64%)	222 (68.5%)	63 (52.1%)		
More than 500	23 (5.2%)	15 (4.8%)	8 (6.6%)		
Featured Usage: 2- Interaction usage			
Sending private message				4.98	0.551^b^
Never	61 (13.7%)	44 (13.6%)	17 (14.0%)		
Multiple times a day	122 (27.4%)	92 (28.3%)	30 (24.9%)		
Updating status				12.601	0.051^b^
Never	126 (28.3%)	90 (27.8%)	36 (29.7%)		
Multiple times a day	16 (3.6%)	11 (3.4%)	5 (4.1%)		
Visiting profiles				7.282	0.07^b^
Never	91 (20.4%)	70 (21.6%)	21 (17.4%)		
Multiple times a day	22 (4.9%)	16 (4.9%)	6 (4.9%)		
Comment on others' notes or photos				12.223	0.057^a^
Never	102 (22.9%)	75 (23.1%)	27 (22.4%)		
Multiple times a day	26 (5.8%)	17 (5.3%)	9 (7.4%)		
Sharing or re-send others' profiles				9.415	0.152^a^
Never	106 (23.8%)	82 (25.3%)	24 (19.8%)		
Multiple times a day	38 (8.6%)	24 (7.4%)	14 (11.6%)		
Checking others' comments or message on your profiles				4.466	0.614^a^
Never	156 (35.1%)	117 (36.1%)	39 (32.2%)		
Multiple times a day	36 (8.1%)	21 (6.3%)	15 (12.4%)		
Featured Usage: 3- Display usage				
Writing notes/blogs				7.007	0.319^b^
Never	122 (27.4%)	90 (27.8%)	32 (26.4%)		
Multiple times a day	15 (3.4%)	12 (3.7%)	3 (2.6%)		
Posting photos				8.676	0.188^b^
Never	167 (37.5%)	127 (39.2%)	40 (33.0%)		
Multiple times a day	10 (2.2%)	6 (1.9%)	4 (3.4%)		
Updating profile image				11.597	0.072^b^
Never	104 (23.4%)	68 (21.0%)	36 (29.7%)		
Multiple times a day	6 (1.3%)	2 (0.6%)	4 (3.3%)		
Affective use when using SNSs: Unhappiness				17.661	0.007*^b^
Never	64 (14.4%)	45 (13.9%)	19 (15.7%)		
Always	17 (3.8%)	8 (2.5%)	9 (7.4%)		
Happiness				19.899	0.092^a^
Never	30 (6.7%)	17 (5.2%)	13 (10.7%)		
Always	45 (10.2%)	33 (10.2%)	12 (9.9%)		
Depression				8.331	0.216^b^
Never	119 (26.7%)	91 (28.1%)	28 (23.1%)		
Always	15 (3.4%)	11 (3.4%)	4 (3.3%)		
Joy				7.723	0592^a^
Never	33 (7.4%)	19 (5.9%)	14 (11.6%)		
Always	47 (10.6%)	32 (9.9%)	15 (12.5%)		
Angry				10.513	0.102^b^
Never	80 (18.0%)	61 (18.8%)	19 (15.7%)		
Always	16 (3.6%)	9 (2.9%)	7 (5.9%)		
Contentment				7.324	0.292^a^
Never	31 (7.0%)	19 (5.8%)	12 (9.9%)		
Always	50 (11.2%)	35 (10.9%)	15 (12.4%)		
Anxiety				7.53	0.224^b^
Never	101 (22.7%)	81 (25.0%)	20 (16.5%)		
Always	18 (4.1%)	12 (3.7%)	6 (5.0%)		
Cheer				4.372	0.626^a^
Never	64 (14.4%)	41 (12.6%)	23 (19.0%)		
Always	41 (9.2%)	30 (9.4%)	11 (9.2%)		
Addiction of SNSs:				0.16	0.900^a^
Non-Disordered users	355 (79.8%)	258 (79.6%)	97 (80.2%)		
Disordered users (SMD)	90 (20.2%)	66 (20.4%)	24 (19.8%)		
Motivation Score	35	34	37	13238.0	0.000^c^
Median (Range)	(14–53)	(14–53)	(20–53)		
SNSs use change from pre-pandemic stage to current time				30.015	0.001^a^
Yes, I navigate it less than before	112 (25.2%)	70 (21.6%)	42 (34.7%)		
No change	223 (50.1%)	188 (58.0%)	35 (28.9%)		
Yes, I navigate it more than before	110 (24.7%)	66 (20.4%)	44 (36.4%)		

^a^Chi-square test, ^b^Monte-Carlo corrected p-value, ^c^Mann Whitney U-test, and ^*^ p <0.05.

Basic, interactive, self-display, and featured SNSs usage as a whole showed a mild positive correlation with the EAT-26 score (rs = 0.135, 0.196, 0.178, and 0.106; *p* = 0.004, 0.034, 0.029, and 0.026, respectively). There was a mild positive association between basic, interactive, self-display, and featured SNSs use. Participants' general tendencies to use SNSs for information seeking, self-status seeking, and socializing to fulfill specific gratifications related to weight loss/dieting, fitness/exercise, cooking, fashion, and bariatric surgeries showed significant positive correlations with the EAT-26 score (rs = 0.178, 0.173, 0.265, all *p* < 0.001). Similarly, the motivation score showed a significant positive correlation with the EAT-26 score (*r* = 0.220, *p* < 0.001). Positive affective SNSs use showed a significant positive correlation with the DEBs score (rs = 0.187, *p* < 0.001). In contrast, negative affective SNSs use showed a significant negative correlation with the EAT-26 score (rs = −0.115, *p* = 0.015, Table 4).

**Table 4:** Spearman's rank correlation matrix of the bivariate variables in relation to EAT-26 score.

		**EAT 26 Score**	**Basic use**	**Interactiveuse**	**Self-display use**	**Featured use**	**Positive affective use**	**Negative affective use**	**Dependence score**	**Motivation score**	**Total MET minutes per week**	**SNSs information seeking use**	**SNSs self- status seeking use**	**SNSs socialization use**
EAT 26 Score	r_s_	1.000	0.135**	0.196*	0.178*	0.106*	0.187**	−0.115*	-0.017	0.220**	0.123**	0.178**	0.173**	0.265**
	*p*	.	0.004	0.034	0.029	0.026	0.000	0.015	0.716	0.000	0.009	0.000	0.000	0.000
Basic use	r_s_	0.135**	1.000	0.213**	0.228**	0.754**	0.129**	0.070	0.093	0.094*	0.088	0.049	-0.007	0.063
	*p*	0.004	.	0.000	0.000	0.000	0.006	0.143	0.051	0.047	0.063	0.307	0.878	0.182
Interactive use	r_s_	0.196*	0.213**	1.000	0.606**	0.924**	0.204**	0.132**	0.157**	0.275**	0.068	0.115*	0.235**	0.189**
	*p*	0.034	0.000	.	0.000	0.000	0.000	0.005	0.001	0.000	0.153	0.015	0.000	0.000
Self-display use	r_s_	0.178*	0.228**	0.606**	1.000	0.776**	0.139**	0.096*	0.072	0.245**	0.105*	0.083	0.207**	0.212**
	*p*	0.029	0.000	0.000	.	0.000	0.003	0.043	0.128	0.000	0.027	0.081	0.000	0.000
Featured use	r_s_	0.106*	0.754**	0.924**	0.776**	1.000	0.215**	0.143**	0.144**	0.283**	0.100*	0.108*	0.226**	0.207**
	*p*	0.026	0.000	0.000	0.000	.	0.000	0.003	0.002	0.000	0.035	0.023	0.000	0.000
Positive affective use	r_s_	0.187**	0.129**	0.204**	0.139**	0.215**	1.000	0.166**	0.399**	0.063	-0.053	0.147**	0.108*	−0.045
	*p*	0.000	0.006	0.000	0.003	0.000	.	0.000	0.000	0.184	0.260	0.002	0.023	0.345
Negative affective use	r_s_	−0.115*	0.070	0.132**	0.096*	0.143**	0.166**	1.000	0.163**	−0.055	0.019	0.006	-0.075	−0.234**
Dependence	*p*	0.015	0.143	0.005	0.043	0.003	0.000	.	0.001	0.248	0.696	0.893	0.114	0.000
score	r_s_	−0.017	0.093	0.157**	0.072	0.144**	0.399**	0.163**	1.000	0.100*	-0.094*	0.149**	0.138**	0.037
	*p*	0.716	0.051	0.001	0.128	0.002	0.000	0.001	.	0.035	0.047	0.002	0.003	0.431
Motivation score	r_s_	0.220**	0.094*	0.275**	0.245**	0.283**	0.063	−0.055	0.100*	1.000	0.079	0.169**	0.219**	0.209**
	*p*	0.000	0.047	0.000	0.000	0.000	0.184	0.248	0.035	.	0.096	0.000	0.000	0.000
Total MET minutes per week	r_s_	0.123**	0.088	0.068	0.105*	0.100*	-0.053	0.019	-0.094*	0.079	1.000	0.085	0.083	0.154**
	*p*	0.009	0.063	0.153	0.027	0.035	0.260	0.696	0.047	0.096	.	0.074	0.080	0.001
SNSs Information seeking use	r_s_	0.178**	0.049	0.115*	0.083	0.108*	0.147**	0.006	0.149**	0.169**	0.085	1.000	0.566**	0.242**
	*p*	0.000	0.307	0.015	0.081	0.023	0.002	0.893	0.002	0.000	0.074	.	0.000	0.000
SNSs Self- status Seeking use	r_s_	0.173**	-0.007	0.235**	0.207**	0.226**	0.108*	−0.075	0.138**	0.219**	0.083	0.566**	1.000	0.335**
	*p*	0.000	0.878	0.000	0.000	0.000	0.023	0.114	0.003	0.000	0.080	0.000	.	0.000
SNSs Socialization use	r_s_	0.265**	0.063	0.189**	0.212**	0.207**	-0.045	−0.234**	0.037	0.209**	0.154**	0.242**	0.335**	1.000
	p	0.000	0.182	0.000	0.000	0.000	0.345	0.000	0.431	0.000	0.001	0.000	0.000	.

**Correlation is significant at the 0.01 level (2-tailed).

*Correlation is significant at the 0.05 level (2-tailed).

p-value is illustrated in italic format.

SNSs, Social Networking Sites ; Total MET, Total metabolic equivalents minutes.

Table 5 spots the factors, which significantly contribute to DEBs. Female students who navigated SNSs sites more in the current time compared to that in the pre-pandemic time were more prone to develop DEBs (adjusted OR = 4.225, 95% CI = 3.114–5.446, *p* < 0.001). Participants who had higher numbers of friends on SNSs and those who visited their SNSs once or for more than an hour were more likely to develop DEBs. The participants who were more likely to develop DEBs reported high motivation scores to use SNSs, marked body image concern, and a general tendency of information-seeking SNSs use [(adjusted OR = 5.032, 95% CI = (3.677–6.432), *p* < 0.001), (adjusted OR = 6.034, 95% CI = (4.791–16.097), *p* = 0.003) and (adjusted OR = 2.130, 95% CI = 2.048–3.219, *p* = 0.001), respectively]. On the other hand, regular drug use seemed to be a preventive factor against DEBs (adjusted OR = 0.277, 95% CI = 0.106–0.726, *p* = 0.009). Self-status seeking SNSs use was found to be a border line factor (adjusted OR = 0.933, 95% CI = 0.871–0.999, *p* = 0.045).

**Table 5:** Regression analysis of usage patterns of social networking sites and disordered eating behaviors among the female university students.

	**Wald**	**df**	**Sig**.	**Odds ratio**	**95% C.I. for Odds Ratio**	
					**Lower**	**Upper**
Do you use drugs on regular basis?	6.814	1	0.009**	0.277	0.106	0.726
SNSs Habits change from pre-pandemic stage to current time	21.069	2	0.000**			
No Change (1)	0.150	1	0.699	0.864	0.413	1.809
Yes, navigate more than before (2)	18.299	1	0.000**	4.225	3.114	5.446
In your favorite SNSs, how many friends do you have?	14.174	6	0.028*			
50–Less than 100 (1)	0.643	1	0.423	0.598	0.170	2.100
100–Less than 200 (2)	0.102	1	0.342	1.240	0.331	4.637
200–Less than 300 (3)	0.818	1	0.366	0.469	0.091	2.419
300–Less than 400 (4)	3.183	1	0.467	0.407	0.036	4.589
400–Less than 500 (5)	1.509	1	0.030*	1.240	1.331	4.637
More than 500 (6)	0.528	1	0.025*	4.260	2.867	20.937
On average, each time you visit SNS, how long would you spend on it?	21.411	6	0.002**			
More than 4 h (1)	6.825	1	0.199	0.538	0.208	1.387
3–4 h (2)	0.714	1	0.398	0.615	0.200	1.897
2–3 h (3)	6.668	1	0.010*	0.195	0.057	0.675
1–2 h (4)	8.857	1	0.003**	0.139	0.038	0.510
0.5–1 h (5)	8.658	1	0.003**	0.123	0.030	0.496
15–30 min (6)	11.229	1	0.001**	0.165	0.058	0.473
Motivation score	12.307	1	0.000**	5.032	3.677	6.432
Body image concern	16.581	4	0.002**			
Mild Concern (1)	0.415	1	0.519	1.368	0.527	3.548
Moderate Concern (2)	5.716	1	0.015*	3.275	2.145	8.179
Marked Concern (3)	8.837	1	0.003**	6.034	4.791	16.097
Information seeking SNSs use	10.114	1	0.001**	2.130	2.048	3.219
Self-status seeking SNSs use	4.008	1	0.045*	0.933	0.871	0.999
Constant	4.002	1	0.045	0.061		

Logistic Regression: Outcome: Eating Disorder (Chi Square X^2^= 158.071, *p* < 0.05*, *p* < 0.001**).

Predictors: Do you have any Psychological Disease? (Reference: No), Do you use drugs on regular basis? (Reference: No), Do any of your family member suffer from Obesity? (Reference: No), BMI in Kg/m2, SNSs Habits change from pre-pandemic stage to current time (Reference: Yes, navigate less than before), 1-How frequently do you use SNSs? (Reference: Never), 3- In your favorite SNSs, how many friends do you have? (Reference: Less than 50), 2-On average, each time you visit SNS, how long would you spend on it? (Reference: 15 min or less), Affective use: Unhappiness, Motivation score, Total MET in minutes per week, featured use, Positive affective use, Negative affective use, Information seeking SNSs use, Self-status seeking SNSs use, SNSs use for socializing, Body image concern (Reference: No).

## Discussion

This study investigated the prevalence of DEBs among Saudi female college students during the COVID-19 pandemic, as well as its association with students' lifestyle behaviors and SNSs use. More than a quarter of the participants (27.2%) had DEBs (Table 1). This finding contradicts those of a recently published study of Saudi college female students, which reported slightly higher rates of DEBs during the COVID-19 pandemic (11). This disparity may be explained by the timing of data collection, differences in population characteristics, and population resilience with the spread of the pandemic. The early stages of the COVID-19 pandemic were frequently associated with elevated levels of distress, depression, and anxiety, and a longitudinal follow-up revealed clear indications of resilience (50). Previous studies have reported more differences in distress, internet use, and eating behaviors among medical students as compared to those in other theoretical majors (51, 52). Nevertheless, a meta-analysis of different studies in Middle Eastern countries published in 2021 reported a slightly lower prevalence of DEBs (22.07%) compared to the current estimated prevalence, which is slightly higher than the global prevalence, owing to rapid social changes and acculturation occurring in the Arab world (7). Some studies conducted prior to the pandemic on Saudi female university students using the EAT-26 test found higher rates of DEBs than those reported in the current study (35.4%, 38.8%, and 29.4%) (8, 9, 14), whereas others showed slightly lower rates (25.4%) (10). Abd El-Azeem et al. (8) found that 35.4% of the 1,200 females at Taif, were at risk for EDs. These differences could be attributed to the timing of the study and various methodological factors such as study setting (university or school, or different cultures in different regions of Saudi Arabia), sample selection (gender and age), size, and assessment methods (self-reported or interviews).

The current study highlights the relationship between the patterns of SNSs use and DEBs. Students who navigate SNSs more frequently in the current time than in the pre-pandemic period were more likely to develop DEBs. Additionally, DEBs are significantly more prevalent among those with higher basic (higher frequency rate of account checking, duration of use, and number of friends on different SNSs), interactive, and self-display use rather than among those with higher addictive SNSs use (Tables 3–5). This finding aligns with the results of a recently published meta-analysis and several previous studies (22, 53, 54). Holland et al. (55), reported that specific actions on SNSs (such as viewing and uploading images, receiving negative comments *via* status updates, and making comments on other SNSs users' photos and statuses) were related to a higher drive for thinness, appearance comparison, and eating concerns. Body dissatisfaction may influence DEBs when SNSs are used. Based on the descriptive statistics of the main domains of EAT-26 tool, we concluded that the university students depicted a high level of oral control compared to the other two domains of DEBs as illustrated in Supplementary Table S1, this may be attributed to the desire to be slimmer. Exposure to media messages advocating a thin ideal body has been linked to disturbances in body image and DEBs. Murray et al. (56) illustrated that body esteem indicators mediate the relationship between SNSs use and EDs. In addition, greater SNSs use was associated with more weight gain and body dissatisfaction, which is associated with more severe EDs. Cohen et al. (57) demonstrated that engaging in photo-based activities (e.g., posting and sharing photos of oneself and friends) rather than general SNSs use was associated with EDs.

Almuhlafi et al. (58) found that 62% of 399 adolescent females in the city of Tabuk, northern Saudi Arabia, believed that social media exposure to fashion designs/modeling industry increased their desire to lose weight. More than half the individuals who felt pressure to be thin and those who thought social media influencers inspired them to work out reported signs of EDs, according to Al-Jumayan et al. (59). In contrast, other studies have reported that SNSs use was not directly related to DEBs (23). Ferguson et al. (60), who measured the impacts of SNSs use and peer competition on body satisfaction and EDs symptoms among teenage girls over a 6-month follow-up period. They found no concurrent or prospective correlations between SNSs use and body dissatisfaction or EDs symptoms. This absence of a relationship could be attributed to the variations in SNSs use measurement, which included activities like online gaming and blogging or the fact that the majority of the participants were Latino (94.1%) (60). Different cultures have different traditional aesthetics. White American adolescents strongly internalize the concept that “beauty is thin.” Therefore, studies on this subject from different regions may yield different findings (61).

In line with findings in the correlation analysis (Table 4), Easton et al. (62) found that viewing Fitspiration posts encouraged participants' obsession with calorie counting, and a some of them reported that some diet-related materials could even instigate EDs, particularly if the participants were unaware that they were developing unhealthy eating habits. In fact, the contents of some diets can induce DEBs (63). Likewise, Lee et al. (64) illustrated that social media use for body image information was negatively correlated with body satisfaction, and, thus, negatively affected DEBs (21). In accordance with the present study, Lee et al. (64) presented their participants with profile pictures of underweight or overweight users on Facebook. They discovered that Korean undergraduates who observed an underweight peer making online comments about wanting to lose weight were less satisfied with their bodies than those who observed an overweight peer expressing the same desire (64).

Previous studies have focused on the impact of SNS activity on well-being; nevertheless, they rarely consider the potential effects of SNS affective experiences that may predict psychological well-being (31). Regardless of individual's activities on SNSs, users are more likely to be satisfied and happier when they experience more positive and fewer negative affective experiences. Fear of not receiving comments/likes (i.e., online neglect) or the fear of receiving negative comments might trigger stressful experiences and negative feelings, resulting in decreased life satisfaction or poor psychological well-being. We found that unhappiness and negative affective experiences were significantly associated with DEBs (Tables 3–5), which is consistent with the results of Fabris et al. (65), who concluded that adolescents with higher levels of negative affective experiences might be at a greater risk for excessive social media use aimed at restoring gratification or compensation with respect to perceived needs, and, accordingly, may increase the probability of psychological consequences and disordered eating among the young population (22, 65). On the other hand, some females with EDs may develop internet addiction while they try to get dieting/weight control information or get social media support from people with similar problems (51, 63).

Previous studies among female university students found significant relationships between EAT-26 scores exceeding 20 and peer or family stress due to losing weight, marital status, studying in health science colleges, positive psychological illness history, overweight or obesity, poor eating habits, vegetarianism, and high levels of PA (8–12). A study conducted on 399 adolescent females in the city of Tabuk, northern Saudi Arabia, found high rates of overweight/obesity and DEBs, and participants with DEBs experienced more significant peer pressure to lose weight (58). Dooley-Hash et al. (66) found a correlation between EDs and depression in females, which is consistent with the reported association between poor psychiatric states and disordered eating. Psychiatric distress triggers emotional eating and unhealthy food choices as a coping mechanism (67). Nevertheless, the current study did not detect a statistically significant difference between DEBs groups regarding faculty type, family and peer support, diet, smoking, sleeping habits, and overall composite lifestyle score. Consistent with the results of Alwosaifer et al. (9), the current study found no significant risk among different academic majors. This could be because all the college students may have experienced similar consequences. Badrasawi et al. also showed that ED risk was not correlated with fast-food consumption, which is consistent with our results (68). The only significantly associated lifestyle behavior was PA, where DEBs is more prevalent among those practicing high PA, as reported in previous studies (59, 69). In 2021, Al-Jumayan et al. conducted a cross-sectional study of 560 sports center clients in Saudi Arabia. They found that exercise frequency was significantly associated with the risk of EDs; higher rates were reported in participants who exercised more frequently per week. DEBs frequency and the need for referral to mental health professionals were found more in participants who reported spending more time in the gym (59). This finding can be attributed to the fact that athletes are more likely to develop DEBs and exercise behaviors because of the pressure to perform well and acquire a specific appearance (70, 71). Our results also revealed that the probability of being overweight or obese, having body image concerns, or having a family history of obesity was relatively high in the DEBs-risk group, which is consistent with many published studies (6, 8, 10). These findings could be explained by psychological co-occurrences of high BMI, such as body concern or dissatisfaction, and weight stigma, thereby contributing to the increasing burden of DEBs (72). Concerns about body image are thought to be a risk factor for DEBs.

This is the first study to examine the relationship between SNSs and DEBs, considering unhealthy lifestyle behaviors during the COVID-19 pandemic. The strengths of this study include the calculation of the sample size, the recruitment of a random sample from various faculties, and the use of validated questionnaires. Additionally, college-aged individuals were targeted because DEBs tend to be more prevalent during this life stage. However, there are some limitations. Data are self-reported, which is susceptible to inevitable recall bias. Second, because of the cross-sectional, we could not infer causal relationships and could not investigate the effect of the COVID pandemic stage on SNSs navigation rate over time. Third, the results are not representative of female university students in Saudi Arabia as a whole but of only one university. Consequently, the findings cannot be generalized to other contexts. Using self-reported weight and height to calculate BMI may result in an underestimation bias, even though previous studies found few differences between self-reported and measured BMIs in females and men. Longitudinal studies are recommended to assess causality between SNSs use and DEBs.

## Conclusion

DEBs are prevalent among female college students, particularly those who use SNSs excessively and have more friends on SNSs. The likelihood of developing DEBs is higher among females who report a high propensity for information-seeking SNSs use, high motivation for SNSs use in relation to weight loss/dieting, and significant body image concerns. Health practitioners need to screen young females for DEBs, necessitating early interventions to treat and prevent potentially harmful consequences. In addition, it is crucial to monitor adolescents' use of the internet, especially social media, which represents a significant risk factor for DEBs.

## Data availability statement

The original contributions presented in the study are included in the article/Supplementary material, further inquiries can be directed to the corresponding author.

## Ethics statement

This study was conducted according to the guidelines laid down in the Declaration of Helsinki and all procedures involving research study participants were approved by the Research Ethics Committee of Taif Health Affairs, Ministry of Health, Saudi Arabia (IRB. HAP-02-T-067, 653). The patients/participants provided their written informed consent to participate in this study.

## Author contributions

AR and NAb designed the study proposal and questionnaire and collected research data. NO and NAb analyzed data. NAb, AR, AMA, and NO shared in data interpretation, drafting the work, and article writing. All authors contributed to the article and approved the submitted version.

## Conflict of interest

The authors declare that the research was conducted in the absence of any commercial or financial relationships that could be construed as a potential conflict of interest.

## Publisher's note

All claims expressed in this article are solely those of the authors and do not necessarily represent those of their affiliated organizations, or those of the publisher, the editors and the reviewers. Any product that may be evaluated in this article, or claim that may be made by its manufacturer, is not guaranteed or endorsed by the publisher.
