# Supplementary material for: The relationship between the high-risk disordered eating and social network navigation among Saudi college females during the COVID pandemic
Source: Front Public Health. 2022 Sep 6;10:949051. doi: 10.3389/fpubh.2022.949051 (PMC9486004; doi:10.3389/fpubh.2022.949051)
Supplement: Supplementary file 1 [file Data_Sheet_1.docx]

**Supplementary Table: (S1): Distribution of the studied university students according to their responses on items of** **Eating Attitude Test (EAT-26):**

|  | **Responses on EAT-26** | **Never/**  **Rarely** | **Sometimes** | **Usually** | **Always** |
| --- | --- | --- | --- | --- | --- |
|  |  | **No. (%)** | **No. (%)** | **No. (%)** | **No. (%)** |
| **Dieting subscale** | I am terrified about being overweight. | 241 (54.2%) | 57 (12.8%) | 46 (10.3%) | 101 (22.7%) |
|  | Aware of the calorie content of foods that I eat. | 347 (78.0%) | 32 (7.2%) | 32 (7.2%) | 34 (7.6%) |
|  | Particularly avoid food with high carbohydrate content (i.e. bread, rice, potatoes.) | 376 (84.5%) | 36 (8.1%) | 19 (4.3%) | 14 (3.1%) |
|  | Feel extremely guilty after eating | 348 (78.2%) | 36 (8.1%) | 19 (4.3%) | 42 (9.4%) |
|  | Am preoccupied with a desire to be thinner. | 288 (64.7%) | 45 (10.1%) | 39 (8.8%) | 73 (16.4%) |
|  | Think about burning up calories when I exercise. | 257 (57.8%) | 50 (11.2%) | 36 (8.1%) | 102 (22.9%) |
|  | Am preoccupied with the thought of having fat on my body. | 326 (73.3%) | 45 (10.1%) | 25 (5.6%) | 49 (11.0%) |
|  | Avoid foods with sugar in them. | 342 (76.9%) | 46 (10.3%) | 38 (8.5%) | 19 (4.3%) |
|  | Eat diet foods. | 369 (83.0%) | 44 (9.9%) | 22 (4.9%) | 10 (2.2%) |
|  | Feel uncomfortable after eating sweets. | 312 (70.1%) | 54 (12.1%) | 28 (6.3%) | 51 (11.5%) |
|  | Engage in dieting behavior. | 379 (85.2%) | 25 (5.6%) | 25 (5.6%) | 16 (3.6%) |
|  | Like my stomach to be empty. | 348 (78.2%) | 46 (10.3%) | 26 (5.8%) | 25 (5.6%) |
|  | Enjoy trying new rich foods | 154 (34.6%) | 49 (11.0%) | 82 (18.4%) | 160 (36.0%) |
| **Bulimia and Food Preoccupation subscale** | Find myself preoccupied with food. | 314 (70.6%) | 62 (13.9%) | 36 (8.1%) | 33 (7.4%) |
|  | Have gone on eating binges where I feel that I may not be able to stop. | 364 (81.8%) | 32 (7.2%) | 29 (6.5%) | 20 (4.5%) |
|  | Vomit after I have eaten. | 413 (92.8%) | 19 (4.3%) | 6 (1.3%) | 7 (1.6%) |
|  | Feel that food controls my life. | 299 (67.2%) | 57 (12.8%) | 38 (8.5%) | 51 (11.5%) |
|  | Give too much time and thought to food. | 352 (79.1%) | 44 (9.9%) | 24 (5.4%) | 25 (5.6%) |
|  | Have the impulse to vomit after meals | 391 (87.9%) | 24 (5.4%) | 13 (2.9%) | 17 (3.8%) |
| **Oral Control Subscale** | Avoid eating when I am hungry. | 347 (78.0%) | 47 (10.6%) | 30 (6.7%) | 21 (4.7%) |
|  | Cut my food into small pieces | 281 (63.1%) | 62 (13.9%) | 49 (11.1%) | 53 (11.9%) |
|  | Feel that others would prefer if I ate more. | 292 (65.6%) | 51 (11.5%) | 18 (4.0%) | 84 (18.9%) |
|  | Other people think that I am too thin. | 274 (61.6%) | 67 (15.1%) | 30 (6.7%) | 74 (16.6%) |
|  | Take longer than others to eat my meals. | 280 (62.9%) | 69 (15.5%) | 30 (6.7%) | 66 (14.9%) |
|  | Display self-control around food. | 219 (49.2%) | 90 (20.2%) | 65 (14.6%) | 71 (16.0%) |
|  | Feel that others pressure me to eat. | 319 (71.7%) | 41 (9.2%) | 37 (8.3%) | 48 (10.8%) |

**Supplementary Table: (S2): Social Network Sites use (Featured Use, Affective use, and addiction of SNSs) among female university students, classified according to EAT-26:**

|  | Total | EAT – 26 [n.199] | | Statistical  test | *p* value |
| --- | --- | --- | --- | --- | --- |
|  | N=445 | Normal | At Risk |  |  |
|  |  | (n =324 ,72.8%) | (n=121, 27.2%) |  |  |
| Holding SNS account (year) | | | | 5.303 | 0.258^a^ |
| Less than two years | 34 (7.6%) | 23(7.1%) | 11(9.1%) |  |  |
| 3-6 year | 135(30.3%) | 106(32.7%) | 29(24.0%) |  |  |
| 7-10 year | 172(38.7%) | 122(37.7%) | 50(41.3%) |  |  |
| More than 10 years | 104(23.4%) | 73(22.5%) | 31(25.6%) |  |  |
| Featured Usage: 1- Basic SNSs usage factor | |  |  |  |  |
| SNS account check (times) | | | | 14.942 | 0.034*^b^ |
| Never | 10(2.2%) | 5(1.5%) | 5(4.1%) |  |  |
| Yearly | 8(1.8%) | 3(0.9%) | 5(4.1%) |  |  |
| Monthly | 5(1.2%) | 3(0.9%) | 2(1.6%) |  |  |
| Weekly | 8(1.8%) | 5(1.6%) | 3(2.5%) |  |  |
| Multiple times a week | 10(2.3%) | 9(2.8%) | 1(0.8%) |  |  |
| Daily | 6(1.3%) | 6(1.8%) | 0(0.0%) |  |  |
| Multiple times a day | 95(21.3%) | 76(23.5%) | 19(15.7%) |  |  |
| Extreme use (once or more an hour) | 303(68.1%) | 217(67.0%) | 86(71.0%) |  |  |
| Duration of using SNSs | | | | 17.257 | 0.008*^b^ |
| 15min or less | 39(8.8%) | 31(9.6%) | 8(6.6%) |  |  |
| 15- 30min | 51(11.5%) | 34(10.5%) | 17(14.0%) |  |  |
| 0.5-1h | 72(16.2%) | 59(18.2%) | 13(10.8%) |  |  |
| 1-2h | 57(12.8%) | 41(12.6%) | 16(13.2%) |  |  |
| 2-3h | 45(10.1%) | 38(11.7%) | 7(5.8%) |  |  |
| 3-4h | 43(9.6%) | 35(10.8%) | 8(6.7%) |  |  |
| More than 4h | 138(31.0%) | 86(26.6%) | 52(42.9%) |  |  |
| Number of friends | | |  | 19.868 | 0.003*^b^ |
| 1 -<50 | 285(64%) | 222(68.5%) | 63(52.1%) |  |  |
| 50 - <100 | 65(14.6%) | 43(13.2%) | 22(18.2%) |  |  |
| 100 - <200 | 29(6.5%) | 23(7.2%) | 6(4.9%) |  |  |
| 200 - <300 | 20(4.5%) | 9(2.6%) | 11(9.1%) |  |  |
| 300 - <400 | 15(3.4%) | 7(2.1%) | 8(6.6%) |  |  |
| 400 - <500 | 8(1.8%) | 5(1.6%) | 3(2.5%) |  |  |
| More than 500 | 23(5.2%) | 15(4.8%) | 8(6.6%) |  |  |
| Featured Usage: 2- Interaction usage | | |  |  |  |
| Sending private message | | |  | 4.98 | 0.551 ^b^ |
| Never | 61(13.7%) | 44(13.6%) | 17(14.0%) |  |  |
| Yearly | 12(2.7%) | 8(2.5%) | 4(3.3%) |  |  |
| Monthly | 36(8.1%) | 27(8.3%) | 9(7.4%) |  |  |
| Weekly | 39(8.8%) | 29(8.9%) | 10(8.3%) |  |  |
| Multiple times a week | 85(19.1%) | 66(20.4%) | 19(15.7%) |  |  |
| Daily | 90(20.2%) | 58(18.0%) | 32(26.4%) |  |  |
| Multiple times a day | 122(27.4%) | 92(28.3%) | 30(24.9%) |  |  |
| Updating status | | |  | 12.601 | 0.051 ^b^ |
| Never | 126(28.3%) | 90(27.8%) | 36(29.7%) |  |  |
| Yearly | 75(16.9%) | 55(17.0%) | 20(16.4%) |  |  |
| Monthly | 97(21.8%) | 82(25.3%) | 15(12.3%) |  |  |
| Weekly | 42(9.4%) | 31(9.6%) | 11(9.1%) |  |  |
| Multiple times a week | 62(13.9%) | 39(12.0%) | 23(19.0%) |  |  |
| Daily | 27(6.1%) | 16(4.9%) | 11(9.1%) |  |  |
| Multiple times a day | 16(3.6%) | 11(3.4%) | 5(4.1%) |  |  |
| Visiting profiles | |  |  | 7.282 | 0.07 ^b^ |
| Never | 91(20.4%) | 70(21.6%) | 21(17.4%) |  |  |
| Yearly | 37(8.3%) | 28(8.6%) | 9(7.4%) |  |  |
| Monthly | 83(18.7%) | 65(20.1%) | 18(14.8%) |  |  |
| Weekly | 66(14.8%) | 47(14.5%) | 19(15.7%) |  |  |
| Multiple times a week | 86(19.3%) | 62(19.1%) | 24(19.9%) |  |  |
| Daily | 60(13.6%) | 36(11.2%) | 24(19.9%) |  |  |
| Multiple times a day | 22(4.9%) | 16(4.9%) | 6(4.9%) |  |  |
| Comment on others’ notes or photos | | |  | 12.223 | 0.057 ^a^ |
| Never | 102(22.9%) | 75(23.1%) | 27(22.4%) |  |  |
| Yearly | 31(7.0%) | 22(6.8%) | 9(7.4%) |  |  |
| Monthly | 73(16.4%) | 59(18.2%) | 14(11.6%) |  |  |
| Weekly | 69(15.5%) | 50(15.4%) | 19(15.7%) |  |  |
| Multiple times a week | 89(20%) | 70(21.6%) | 19(15.7%) |  |  |
| Daily | 55(12.4%) | 31(9.6%) | 24(19.8%) |  |  |
| Multiple times a day | 26(5.8%) | 17(5.3%) | 9(7.4%) |  |  |
| Sharing or re-send others’ profiles | | | | 9.415 | 0.152 ^a^ |
| Never | 106(23.8%) | 82(25.3%) | 24(19.8%) |  |  |
| Yearly | 37(8.3%) | 27(8.3%) | 10(8.3%) |  |  |
| Monthly | 65(14.6%) | 50(15.4%) | 15(12.4%) |  |  |
| Weekly | 53(11.9%) | 40(12.4%) | 13(10.7%) |  |  |
| Multiple times a week | 93(20.9%) | 70(21.6%) | 23(19.0%) |  |  |
| Daily | 53(11.9%) | 31(9.6%) | 22(18.2%) |  |  |
| Multiple times a day | 38(8.6%) | 24(7.4%) | 14(11.6%) |  |  |
| Checking others’ comments or message on your profiles | |  |  | 4.466 | 0.614 ^a^ |
| Never | 156(35.1%) | 117(36.1%) | 39(32.2%) |  |  |
| Yearly | 29(6.5%) | 21(6.5%) | 8(6.5%) |  |  |
| Monthly | 54(12.1%) | 39(12.0%) | 15(12.4%) |  |  |
| Weekly | 37(8.3%) | 28(8.6%) | 9(7.4%) |  |  |
| Multiple times a week | 66(14.8%) | 48(14.8%) | 18(15.0%) |  |  |
| Daily | 67(15.1%) | 50(15.4%) | 17(14.1%) |  |  |
| Multiple times a day | 36(8.1%) | 21(6.3%) | 15(12.4%) |  |  |
| Featured Usage: 3- Display usage | |  |  |  |  |
| Writing notes/blogs | |  |  | 7.007 | 0.319 ^b^ |
| Never | 122(27.4%) | 90(27.8%) | 32(26.4%) |  |  |
| Yearly | 34(7.6%) | 26(8.0%) | 8(6.6%) |  |  |
| Monthly | 97(21.8%) | 77(23.8%) | 20(16.5%) |  |  |
| Weekly | 60(13.5%) | 42(13.0%) | 18(14.8%) |  |  |
| Multiple times a week | 81(18.2%) | 51(15.7%) | 30(24.8%) |  |  |
| Daily | 36(8.1%) | 26(8.0%) | 10(8.3%) |  |  |
| Multiple times a day | 15(3.4%) | 12(3.7%) | 3(2.6%) |  |  |
| Posting photos | |  |  | 8.676 | 0.188 ^b^ |
| Never | 167(37.5%) | 127(39.2%) | 40(33.0%) |  |  |
| Yearly | 35(7.9%) | 27(8.3%) | 8(6.6%) |  |  |
| Monthly | 104(23.4%) | 81(25.0%) | 23(19.0%) |  |  |
| Weekly | 52(11.7%) | 34(10.5%) | 18(14.9%) |  |  |
| Multiple times a week | 61(13.7%) | 40(12.3%) | 21(17.3%) |  |  |
| Daily | 16(3.6%) | 9(2.8%) | 7(5.8%) |  |  |
| Multiple times a day | 10(2.2%) | 6(1.9%) | 4(3.4%) |  |  |
| Updating profile image | |  |  | 11.597 | 0.072 ^b^ |
| Never | 104(23.4%) | 68(21.0%) | 36(29.7%) |  |  |
| Yearly | 128(28.8%) | 103(31.8%) | 25(20.7%) |  |  |
| Monthly | 155(34.8%) | 114(35.2%) | 41(33.9%) |  |  |
| Weekly | 21(4.8%) | 15(4.6%) | 6(5.0%) |  |  |
| Multiple times a week | 25(5.6%) | 18(5.6%) | 7(5.8%) |  |  |
| Daily | 6(1.3%) | 4(1.2%) | 2(1.6%) |  |  |
| Multiple times a day | 6(1.3%) | 2(0.6%) | 4(3.3%) |  |  |
| Affective use when using SNSs: Unhappiness | | | | 17.661 | 0.007*^b^ |
| Never | 64(14.4%) | 45(13.9%) | 19(15.7%) |  |  |
| Rarely | 95(21.3%) | 77(23.8%) | 18(14.8%) |  |  |
| Occasionally | 137(30.8%) | 109(33.6%) | 28(23.3%) |  |  |
| Sometimes | 77(17.4%) | 50(15.4%) | 27(22.3%) |  |  |
| Frequently | 38(8.5%) | 23(7.1%) | 15(12.4%) |  |  |
| Usually | 17(3.8%) | 12(3.7%) | 5(4.1%) |  |  |
| Always | 17(3.8%) | 8(2.5%) | 9(7.4%) |  |  |
| Happiness | | | | 19.899 | 0.092 ^a^ |
| Never | 30(6.7%) | 17(5.2%) | 13(10.7%) |  |  |
| Rarely | 46(10.3%) | 28(8.6%) | 18(14.9%) |  |  |
| Occasionally | 82(18.4%) | 64(19.7%) | 18(14.9%) |  |  |
| Sometimes | 108(24.3%) | 79(24.5%) | 29(24.0%) |  |  |
| Frequently | 86(19.3%) | 69(21.3%) | 17(14.0%) |  |  |
| Usually | 48(10.8%) | 34(10.5%) | 14(11.6%) |  |  |
| Always | 45(10.2%) | 33(10.2%) | 12(9.9%) |  |  |
| Depression | | | | 8.331 | 0.216 ^b^ |
| Never | 119(26.7%) | 91(28.1%) | 28(23.1%) |  |  |
| Rarely | 133(29.9%) | 102(31.5%) | 31(25.6%) |  |  |
| Occasionally | 85(19.2%) | 60(18.5%) | 25(20.7%) |  |  |
| Sometimes | 45(10.1%) | 31(9.6%) | 14(11.6%) |  |  |
| Frequently | 26(5.8%) | 18(5.5%) | 8(6.6%) |  |  |
| Usually | 22(4.9%) | 11(3.4%) | 11(9.1%) |  |  |
| Always | 15(3.4%) | 11(3.4%) | 4(3.3%) |  |  |
| Joy | | | | 7.723 | 0592 ^a^ |
| Never | 33(7.4%) | 19(5.9%) | 14(11.6%) |  |  |
| Rarely | 47(10.6%) | 36(11.1%) | 11(9.1%) |  |  |
| Occasionally | 91(20.4%) | 63(19.4%) | 28(23.1%) |  |  |
| Sometimes | 104(23.4%) | 81(25.0%) | 23(19.0%) |  |  |
| Frequently | 77(17.3%) | 60(18.5%) | 17(14.0%) |  |  |
| Usually | 46(10.3%) | 33(10.2%) | 13(10.7%) |  |  |
| Always | 47(10.6%) | 32(9.9%) | 15(12.5%) |  |  |
| Angry | | | | 10.513 | 0.102 ^b^ |
| Never | 80(18.0%) | 61(18.8%) | 19(15.7%) |  |  |
| Rarely | 119(26.7%) | 93(28.7%) | 26(21.5%) |  |  |
| Occasionally | 110(24.7%) | 82(25.3%) | 28(23.1%) |  |  |
| Sometimes | 66(14.8%) | 40(12.3%) | 26(21.5%) |  |  |
| Frequently | 42(9.4%) | 29(8.9%) | 13(10.7%) |  |  |
| Usually | 12(2.8%) | 10(3.1%) | 2(1.6%) |  |  |
| Always | 16(3.6%) | 9(2.9%) | 7(5.9%) |  |  |
| Contentment | | | | 7.324 | 0.292 ^a^ |
| Never | 31(7.0%) | 19(5.8%) | 12(9.9%) |  |  |
| Rarely | 59(13.3%) | 37(11.4%) | 22(18.2%) |  |  |
| Occasionally | 95(21.3%) | 73(22.5%) | 22(18.2%) |  |  |
| Sometimes | 110(24.7%) | 84(26.0%) | 26(21.5%) |  |  |
| Frequently | 51(11.5%) | 39(12.0%) | 12(9.9%) |  |  |
| Usually | 49(11.0%) | 37(11.4%) | 12(9.9%) |  |  |
| Always | 50(11.2%) | 35(10.9%) | 15(12.4%) |  |  |
| Anxiety | | | | 7.53 | 0.224 ^b^ |
| Never | 101(22.7%) | 81(25.0%) | 20(16.5%) |  |  |
| Rarely | 126(28.3%) | 95(29.3%) | 31(25.6%) |  |  |
| Occasionally | 95(21.3%) | 67(20.7%) | 28(23.1%) |  |  |
| Sometimes | 61(13.7%) | 42(13.0%) | 19(15.7%) |  |  |
| Frequently | 28(6.3%) | 18(5.5%) | 10(8.3%) |  |  |
| Usually | 16(3.6%) | 9(2.8%) | 7(5.8%) |  |  |
| Always | 18(4.1%) | 12(3.7%) | 6(5.0%) |  |  |
| Cheer | | | | 4.372 | 0.626 ^a^ |
| Never | 64(14.4%) | 41(12.6%) | 23(19.0%) |  |  |
| Rarely | 68(15.3%) | 50(15.4%) | 18(14.8%) |  |  |
| Occasionally | 101(22.7%) | 78(24.1%) | 23(19.0%) |  |  |
| Sometimes | 95(21.3%) | 72(22.2%) | 23(19.0%) |  |  |
| Frequently | 41(9.2%) | 28(8.6%) | 13(10.7%) |  |  |
| Usually | 35(7.9%) | 25(7.7%) | 10(8.3%) |  |  |
| Always | 41(9.2%) | 30(9.4%) | 11(9.2%) |  |  |
| Addiction of SNSs:  1- How often during the last year have you spent a lot of time thinking about social media or planned use of social media? | | | | 1.905 | 0.753 ^a^ |
| Very rarely | 130(29.2%) | 93(28.7%) | 37(30.6%) |  |  |
| Rarely | 107(24.0%) | 77(23.8%) | 30(24.8%) |  |  |
| Sometimes | 38(8.5%) | 25(7.7%) | 13(10.7%) |  |  |
| Often | 45(10.1%) | 34(10.5%) | 11(9.1%) |  |  |
| Very often | 125(28.2%) | 95(29.3%) | 30(24.8%) |  |  |
| 2- How often during the last year have you felt an urge to use social media more and more? | | | | 2.598 | 0.627 ^a^ |
| Very rarely | 100(22.5%) | 70(21.6%) | 30(24.8%) |  |  |
| Rarely | 104(23.4%) | 77(23.8%) | 27(22.3%) |  |  |
| Sometimes | 42(9.4%) | 28(8.6%) | 14(11.6%) |  |  |
| Often | 57(12.8%) | 40(12.3%) | 17(14.0%) |  |  |
| Very often | 142(31.9%) | 109(33.7%) | 33(27.3%) |  |  |
| 3- How often during the last year have you used social media to forget about personal problems? | | | | 5.371 | 0.251 ^a^ |
| Very rarely | 99(22.2%) | 79(24.4%) | 20(16.5%) |  |  |
| Rarely | 84(18.9%) | 62(19.1%) | 22(18.2%) |  |  |
| Sometimes | 57(12.8%) | 36(11.1%) | 21(17.3%) |  |  |
| Often | 63(14.2%) | 46(14.2%) | 17(14.0%) |  |  |
| Very often | 142(31.9%) | 101(31.2%) | 41(34.0%) |  |  |
| 4- How often during the last year have you How often during the last year have you tried to cut down on the use of social media without success? | | | | 4.117 | 0.39 ^a^ |
| Very rarely | 123(27.6%) | 85(26.2%) | 38(31.4%) |  |  |
| Rarely | 106(23.8%) | 83(25.7%) | 23(19.0%) |  |  |
| Sometimes | 24(5.4%) | 15 (4.6%) | 9(7.4%) |  |  |
| Often | 51(11.5%) | 39(12.0%) | 12(10.0%) |  |  |
| Very often | 141(31.7%) | 102(31.5%) | 39(32.2%) |  |  |
| 5- How often during the last year have you become restless or troubled if you have been prohibited from using social media? | | | | 2.577 | 0.631 ^a^ |
| Very rarely | 129(29.0%) | 89(27.5%) | 40(33.0%) |  |  |
| Rarely | 113 (25.4%) | 87(26.8%) | 26(21.5%) |  |  |
| Sometimes | 38(8.5%) | 26(8.0%) | 12(10.0%) |  |  |
| Often | 50(11.3%) | 38(11.7%) | 12(10.0%) |  |  |
| Very often | 115(25.8%) | 84(26.0%) | 31(25.5%) |  |  |
| 6- How often during the last year have you used social media so much that it has had a negative impact on your job/studies? | | | | 1.549 | 0.816 ^a^ |
| Very rarely | 131(29.4%) | 96(28.6%) | 35(29.0%) |  |  |
| Rarely | 118(26.6%) | 89(25.5%) | 29(24.0%) |  |  |
| Sometimes | 32(7.2%) | 21(6.5%) | 11(9.0%) |  |  |
| Often | 49(11%) | 34(10.4%) | 15(12.4%) |  |  |
| Very often | 115(25.8%) | 84(28.0%) | 31(25.6%) |  |  |
